# Compartmental fat distribution in the abdomen of dogs relative to overall body fat composition

**DOI:** 10.1186/s12917-020-02327-1

**Published:** 2020-03-30

**Authors:** R. B. S. Turner, D. Tyrrell, G. Hepworth, F. R. Dunshea, C. S. Mansfield

**Affiliations:** 1 U-Vet Animal Hospital, Faculty of Veterinary and Agricultural Sciences, University of Melbourne, 250 Princes Highway, Werribee, Victoria 3030 Australia; 2 Statistical Consulting Centre, University of Melbourne, 139 Barry Street, Carlton, Melbourne, Victoria 3053 Australia; 3 Faculty of Veterinary and Agricultural Sciences, University of Melbourne, Parkville, Victoria 3052 Australia

**Keywords:** Body composition, Fat distribution, Visceral fat, Subcutaneous fat, CT, DXA, Dog

## Abstract

**Background:**

Adipose tissue may have different metabolic and endocrine functions depending on the region of the body in which it is located. While visceral or intra-abdominal fat has been found to contribute to leptin concentrations, insulin resistance and obesity-related diseases, there are only a few imaging studies documenting the preferential distribution of body fat to either the intra-abdominal or subcutaneous compartments in dogs. This study aimed to determine if CT-measured abdominal fat distributed preferentially to the visceral space (V) relative to the subcutaneous space (SQ), with increasing DXA-determined total body fat percentage; and if ultrasound measurements of the ventral midline subcutaneous (SAT) and visceral adipose thickness (VAT) can be used to estimate the distribution of fat to the subcutaneous and visceral abdominal spaces, in a sample of 22 dogs with variable body condition.

**Results:**

Multivariate analysis showed no statistically significant correlation between visceral to subcutaneous fat ratio (V/SQ) and increasing total body fat percentage (β = − 0.07, *p* = 0.733), but strong correlation with age (β = 0.71 *p* = 0.002). A substantial amount of variation for the ultrasound visceral adipose thickness to subcutaneous fat thickness (VAT/SAT) could be explained by both CT V/SQ and sex (R^2^_Adjusted_ = 0.477, *p* = 0.001), with female dogs having significant lower VAT/SAT ratios compared to the male dogs (*p* = 0.047). The ultrasound fat measurements appeared moderately reliable, but a larger sample number is required to confirm this.

**Conclusions:**

The findings suggest that dogs with a relatively healthy to slightly overweight body condition score, distribute fat relatively similarly between their peritoneal (visceral) and subcutaneous abdominal compartments with increasing total body fat percentage. However, there was increased fat distribution to the peritoneal space relative to the subcutaneous space with increasing age. Further, abdominal ultrasound may be useful in estimating the ratio of fat distribution to both the abdominal visceral and subcutaneous spaces.

## Background

Obesity is the leading form of malnutrition in dogs, contributing to the pathogenesis of many cardiovascular, metabolic and orthopaedic diseases [1–7]. Further, there is a growing weight of evidence that fat distribution between the visceral and subcutaneous compartments may influence adipokine, insulin and cytokine regulation, and this is related to the development of obesity-related diseases [2, 6–9]. Validating non-invasive, objective and practical means of quantifying fat content and distribution is an essential step in further understanding their effects on the development and outcome of fat-related diseases.

Dual-energy x-ray absorptiometry (DXA) is considered the antemortem “gold standard” in determining body fat content and percentage in dogs, being both highly accurate and reliable at determining body composition [1, 10–14]. However, DXA is currently unable to determine the compartmental distribution of fat within dogs’ abdomens, as the geometric modelling algorithms used in human DXA analysis have not been validated in dogs [15]. Additionally, these human geometric models rely on an assumed distribution of fat within the abdomen, with ethnic variation; and thus, may not apply to various dog conformations [15, 16].

Computed tomography (CT) has overcome many of the limitations encountered by DXA and allows body fat content and distribution to be accurately assessed in dogs [7, 17–20]. Computed tomography offers several advantages over DXA analysis due to its rapid acquisition time, improved spatial and contrast resolution, ability to view structures in three dimensions, and ability to assign a quantitative value to tissues of different attenuation (Hounsfield units). These benefits have allowed researchers to determine fat distribution between the abdominal visceral and subcutaneous compartments and their association with health outcomes such as cardiovascular and metabolic parameters [2, 6, 19]. However, there is limited research on the normal distribution of fat in healthy dogs of mixed breed and age with increasing body fat percentage.

Additionally, CT remains relatively impractical due to its requirement for sedation, and access to equipment and expertise, thus several authors have validated various ultrasonographic measurements of subcutaneous fat to estimate overall body fat content in dogs [21–23]. Ultrasound measurements of subcutaneous fat, particularly lumbar measurements have a strong correlation with total body fat content [21–23]. However, to the authors’ knowledge, there are no ultrasonographic measures that have been validated to estimate fat distribution to the visceral compartment in dogs.

This study aimed to determine if CT-measured abdominal fat distributed preferentially to the peritoneal space (visceral fat) relative to the subcutaneous space, with increasing DXA-determined total body fat percentage; and if ultrasound measurements of the ventral midline subcutaneous and intra-abdominal fat can be used to estimate the distribution of fat to the subcutaneous and visceral abdominal spaces, in a population of dogs with variable body condition.

## Results

### Descriptive statistics

The study population was composed of 22 dogs including 8 neutered females, 3 entire females, 10 castrated males and 1 entire male. The mean age of the dogs was 4.3 years (range of 6 months to 9 years). The mean body weight was 23.4 kg (range of 5.1–60 kg) with a median body condition score of 6 out of 9 (range 4–7) (see Table 1). Male dogs were on average heavier than female dogs in this sample population (*p* = 0.025). All dogs were healthy on physical examination, with no medical history of metabolic disease.

**Table 1:** Summary statistics for body weight, BCS, DXA fat mass, CT tissue and fat distribution measurements, and ultrasound subcutaneous (SAT), visceral (VAT), and total (TAT) adipose thicknesses in dogs (*n* = 22)

Trait	Mean	SD	Min	Max	CV (%)
Body weight (kg)	23.4	13.1	5.1	60.0	56.1
BCS (1–9)	5.7	0.9	4.0	7.0	15.4
DXA total body fat mass (kg)	5.9	3.5	1.7	17.3	58.7
DXA total body fat percentage (%)	27.7	7.6	15.3	48.2	27.3
CT abdominal tissue volume (L)	6.6	3.7	1.5	17.3	55.9
CT abdominal fat volume (L)	2.7	2.0	0.6	9.7	75.2
CT abdominal fat mass (kg)^a^	2.5	1.9	0.6	8.9	75.5
CT abdominal fat percentage (%)^b^	10.6	2.9	5.0	15.2	27.4
CT relative abdominal fat percentage (%)^c^	39.4	6.0	29.9	51.7	15.3
CT visceral fat volume (L)	1.0	0.8	0.2	4.0	82.2
CT visceral fat mass (kg)^a^	0.9	0.8	0.2	3.7	82.8
CT visceral fat percentage (%)^b^	4.0	1.4	1.7	6.7	35.0
CT relative visceral fat percentage (%)^c^	14.6	3.1	10.7	21.2	21.4
CT SQ fat volume (L)	1.7	1.2	0.4	5.7	72.2
CT SQ fat mass (kg)^a^	1.6	1.1	0.3	5.3	72.4
CT SQ fat percentage (%)^b^	6.6	1.7	3.1	9.0	25.8
CT relative SQ fat percentage (%)^b^	24.8	4.4	17.0	32.3	39.0
CT V/SQ Ratio	0.60	0.14	0.36	0.86	23.2
US SAT (mm)	5.5	2.0	2.4	8.9	35.9
US VAT (mm)	13.5	8.1	3.4	34.1	60.2
US TAT (mm)	18.9	9.4	6.0	42.5	49.6
US VAT/SAT	2.51	1.29	0.57	6.05	51.6

*CT* computed tomography, *CV* coefficient of variation, *DXA* dual-energy x-ray absorptiometry, *SAT* subcutaneous adipose thickness, *SD* standard deviation, *SQ* abdominal subcutaneous fat around the abdominal wall, *TAT* total adipose thickness (VAT + SAT), *US* ultrasound, *V/SQ* visceral-to-subcutaneous fat ratio, *VAT* visceral adipose thickness, *VAT/SAT* visceral adipose thickness-to-subcutaneous adipose thickness ratio

^a^ fat mass calculated from fat volume assuming fat density of 0.923 kg/L

^b^ fat percentage calculated by regional fat mass relative to total body mass

^c^ fat percentage calculated by regional fat mass relative to total body fat mass

### Fat distribution

The bivariate linear correlations of CT abdominal fat measurement relative to DXA are listed in Table 2. Both DXA total body fat percentage and age reached the set criteria of a linear relationship (r ≥ 0.3) for both abdominal fat percentage and V/SQ and were included in the multivariate analyses.

**Table 2:** Pearson’s correlations between total body fat percentage (DXA Fat %), age, body weight, BCS and CT measurements of subcutaneous and visceral fat volume in 22 mixed-breed dogs

Trait	Age	Weight	BCS	DXA Fat %	CT AB Fat %	CT Rel AB Fat %	CT V Fat %	CT Rel V Fat %	CT Fat SQ %	CT Rel SQ Fat %	V/SQ
Age		−0.104	0.116	0.533*	0.524*	0.041	0.676**	0.486*	0.341	−0.291	0.676**
Weight			0.213	−0.384	0.078	0.749**	0.038	0.527*	0.102	0.659**	− 0.065
BCS				0.419	0.439*	0.063	0.400	0.113	0.421	0.007	0.156
DXA total body fat percentage (%)					0.818**	−0.153	0.777**	0.106	0.760**	−0.287	0.311
CT total abdominal fat percentage (%)^a^						0.430*	0.924**	0.536*	0.950**	0.211	0.295
CT relative abdominal fat percentage (%)^b^							0.328	0.719**	0.464*	0.869*	−0.059
CT visceral fat percentage (%)^a^								0.692**	0.758**	−0.040	0.624**
CT relative visceral fat percentage (%)^b^									0.348	0.280	0.641
CT SQ fat percentage (%)^a^										0.393	−0.008
CT relative SQ fat percentage (%)^b^											−0.538**

*BCS* body condition score, *CT* computed tomography, *DXA* Dual-energy X-ray absorptiometry, *SQ* abdominal subcutaneous; V/SQ visceral-to-subcutaneous fat ratio

Correlation coefficients with * *p* < 0.05; ** *p* < 0.01

^a^ fat percentage calculated by regional fat mass relative to total body mass

^b^ fat percentage calculated by regional fat mass relative to total body fat mass

**Fig. 1:**
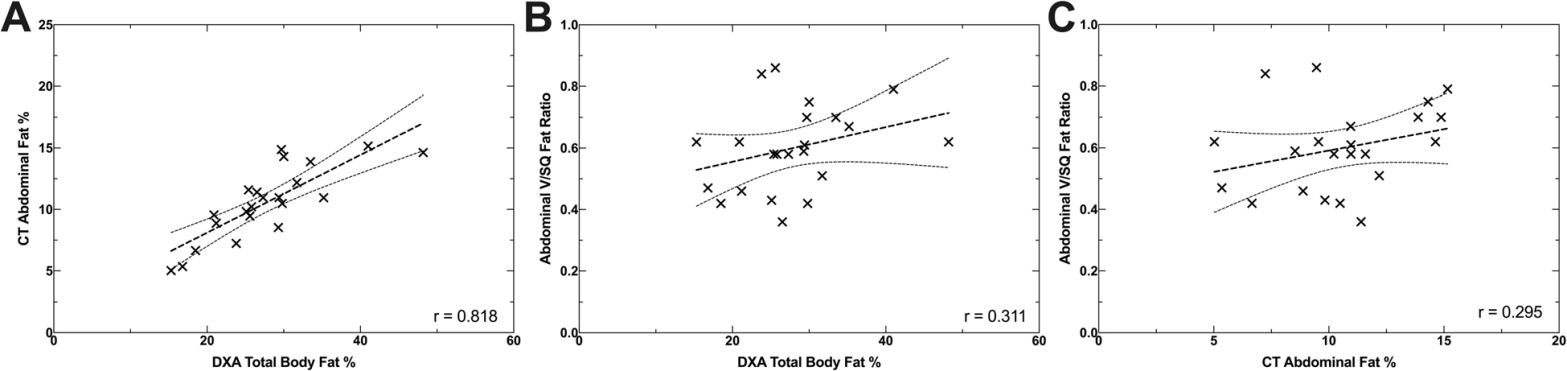
Scatter plots of fat distribution in 22 dogs showing pairwise comparison of CT total abdominal fat percentage relative to DXA total body fat percentage (%) (**a**); CT abdominal visceral fat to subcutaneous fat ratio (V/SQ) relative to DXA total body fat percentage (%) (**b**); and CT abdominal visceral fat to subcutaneous fat ratio relative to CT total abdominal fat percentage (%) (**c**)

Multiple linear regression revealed that DXA total body fat percentage and age explained a substantial proportion of the variation in the value of the CT abdominal fat percentage (R^2^ = 0.680, R^2^_Adjusted_ = 0.647, *p* < 0.001). On its own, age did not significantly predict CT abdominal fat percentage (β = 0.12, *p* = 0.431), however DXA total body fat percentage did significantly predict CT abdominal fat percentage (β = 0.75, *p* < 0.001) (see Fig. 1).

There was no significant difference in mean abdominal fat percentage between the sexes (*p* = 0.074), when controlling for DXA total body fat percentage and age. The combined model explained over half the variation in total abdominal fat percentage (R^2^ = 0.734, R^2^_Adjusted_ = 0.689), though only DXA total body fat was significant (partial η^2^ = 0.627, p < 0.001).

**Fig. 2:**
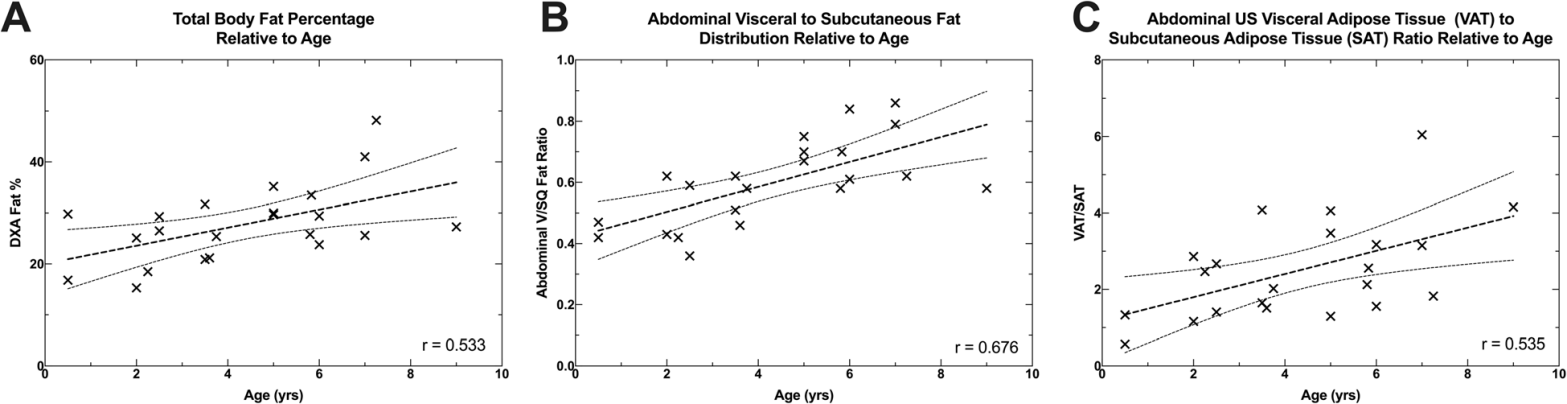
Scatter plots showing pairwise comparison of age relative to DXA fat percentage (%) (**a**); age relative to compartmental fat distribution of the abdomen as measured by CT (V/SQ) (**b**); and age relative to ventral compartmental fat distribution of the abdomen as measured by ultrasound (VAT/SAT) (**c**) in 22 mixed-breed dogs

A multiple linear regression was conducted to see if the ratio of abdominal visceral to subcutaneous fat (V/SQ) could be predicted by DXA total body fat percentage and age. One observation exceeded a Cook’s distance of one (dog 15 had a DXA total body fat of 48.2%), but was not excluded from the analysis. DXA total body fat percentage and age explained nearly half the variation in the V/SQ value (R^2^ = 0.460, R^2^_Adjusted_ = 0.403, *p* = 0.003). On their own, DXA total body fat percentage did not significantly predict V/SQ fat distribution (β = − 0.07, *p* = 0.733), however age did significantly predict V/SQ fat distribution (β = 0.71, *p* = 0.002) (see Fig. 1 and Fig. 2).

The combined model found no significant difference in mean V/SQ between the sexes (*p* = 0.551), when controlling for DXA total body fat percentage and age (see Table 3). The model explained nearly half the variation in V/SQ (R^2^ = 0.471, R^2^_Adjusted_ = 0.383), though only age was significant (partial η^2^ = 0.355, *p* < 0.006).

**Table 3:** Means, adjusted means, standard deviation, standard errors and adjusted 95% confidence interval for sex whilst controlling for DXA total body fat percentage and age (*n* = 22)

	Total Abdominal Fat Percentage		Visceral to Subcutaneous Fat Ratio	
	Male	Female	Male	Female
Mean	11.20	9.90	0.64	0.56
(SD)	(3.23)	(2.57)	(0.14)	(0.13)
Adjusted Mean	11.25	9.86	0.61	0.58
(SE)	(0.51)	(0.51)	(0.03)	(0.03)
Adjusted 95% CI	10.18 to 12.31	8.80 to 10.91	0.54 to 0.69	0.51 to 0.67

*CI* adjusted 95% Confidence Interval

Abdominal fat percentage was not included as a covariate in the V/SQ combined model, as it was not significant (r = 0.295), and a strong association existed with the DXA total body fat percentage (r = 0.818). The neuter status (entire compared to desexed) was excluded from the analysis due to the small sample size (4 entire dogs).

### Ultrasound measurements

The bivariate linear correlations of ultrasound fat measurement relative to DXA and CT fat measurements are listed in Table 4. There was moderate correlation between ultrasound VAT/SAT ratio and the CT V/SQ ratio (r = 0.644) and age (r = 0.535) and poor correlation with DXA total fat percentage (r = 0.133).

**Table 4:** Pearson correlation between age and morphometric fat variables measured by DXA and abdominal CT relative to the ultrasound measurements of subcutaneous and visceral adipose thickness in 22 mixed-breed dogs

Trait	VAT (mm)	SAT (mm)	TAT (mm)	VAT/SAT
Age (yrs)	0.456*	−0.003	0.393	0.535*
Weight (kg)	0.711**	0.568**	0.732**	0.293
BCS (1–9)	0.305	0.200	0.305	0.307
DXA total body fat mass (kg)	0.804**	0.660**	0.831**	0.334
DXA total body fat percentage (%)	0.034	−0.021	0.025	0.133
CT total abdominal fat mass (kg)^a^	0.783**	0.638**	0.809**	0.326
CT total abdominal fat percentage (%)^b^	0.396	0.323	0.409	0.293
CT relative abdominal fat percentage (%)^c^	0.586**	0.622**	0.635**	0.203
CT visceral fat mass (kg)^a^	0.832**	0.594**	0.842**	0.412
CT visceral fat percentage (%)^b^	0.485*	0.163	0.452*	0.515*
CT relative visceral fat percentage %^c^	0.739**	0.348	0.711**	0.598**
CT SQ fat mass (kg)^a^	0.734**	0.656**	0.770**	0.261
CT SQ fat percentage (%)^b^	0.278	0.417	0.327	0.079
CT relative SQ fat percentage %^c^	0.282	0.611**	0.371	−0.146
CT V/SQ	0.396	−0.195	0.301	0.644**

*BCS* body condition score, *CT* computed tomography, *DXA* Dual-energy X-ray Absorptiometry, *SAT* subcutaneous adipose thickness, *SQ* abdominal subcutaneous, *TAT* total adipose thickness, *V/SQ* visceral-to-subcutaneous fat ratio, *VAT* visceral adipose thickness

Correlation coefficient values connected with * *p* < 0.05; ** *p* < 0.01

^a^ fat mass calculated from fat volume assuming fat density of 0.923 kg/L

^b^ fat percentage calculated by regional fat mass relative to total body mass

^c^ fat percentage calculated by regional fat mass relative to total body fat mass

A multiple linear regression was conducted and CT abdominal fat V/SQ and age explained a substantial amount of the variation in the VAT/SAT value (R^2^ = 0.433, R^2^_Adjusted_ = 0.373, *p* = 0.005). In the combined model, CT abdominal fat V/SQ significantly predicted VAT/SAT fat distribution (β = 0.52, *p* = 0.039), but age was not significant (β = 0.19, *p* = 0.440).

A model was fitted to examine the effect of sex on VAT/SAT, whilst controlling for CT V/SQ ratio and age. Age was not significant (*p* = 0.509) and was discarded from the model. Controlling for V/SQ, male dogs (adjusted 95%CI: 2.35 to 3.55) had a significant greater VAT/SAT ratio compared to female dogs (adjusted 95%CI: 1.46 to 2.67; *p* = 0.047). The model explained a significant amount of variation in the VAT/SAT ratio (R^2^ = 0.526, R^2^_Adjusted_ = 0.477, *p* = 0.001).

### Reliability of ultrasound measurements

Unfortunately, only 5 inter-observer measurements were obtained due to work scheduling commitments preventing dual clinic time to perform the measurements.

The small sample size resulted in large confidence intervals, limiting the value of both the calculated intra- and inter-observer variability. However, the ultrasound fat measurements showed moderate reliability and the values are included for comparison (see Table 5).

**Table 5:** Intra-observer and inter-observer intra-class correlation coefficient (95% Confidence Intervals) of ultrasound measurements of the visceral adipose thickness in dogs

	Intra-observer Agreement		Inter-observer Agreement
	Observer 1	Observer 2	
VAT	0.847 (0.463 to 0.981)	0.885 (0.554 to 0.986)	0.839 (−0.111 to 0.982)
SAT	0.848 (0.476 to 0.981)	0.772 (0.333 to 0.970)	0.897 (−0.272 to 0.990)
TAT	0.907 (0.649 to 0.989)	0.533 (0.025 to 0.922)	0.922 (0.181 to 0.992)
VAT/SAT	0.348 (−0.260 to 0.888)	0.718 (0.150 to 0.960)	0.683 (−0.571 to 0.964)

*SAT* subcutaneous adipose thickness, *TAT* total adipose thickness, *V/SQ* visceral-to-subcutaneous fat ratio, *VAT* visceral adipose thickness

Additionally, there was some indication that the VAT measurements differed between expiration (mean = 11.7 mm; SD = 5.2 mm) and inspiration (mean = 9.8 mm; SD = 4.2; 95%CI: − 0.2 to 4.0, *p* = 0.069), as the Cohen’s effect size value (*d* = 0.4) suggested a moderate practical significance.

## Discussion

The major finding from this study was that there is a poor correlation between the preferential distribution of fat to the intra-abdominal compartment (V/SQ) in dogs with increasing total body fat percentage (β = − 0.07, *p* = 0.733). However, the multivariable analyses showed a moderate correlation between age and increasing visceral fat relative to subcutaneous fat around the abdomen in these dogs (age: β = 0.71 *p* = 0.002).

Similar to previous studies, the total abdominal fat strongly correlated to total DXA body fat percentage (r = 0.818) [17, 19]. Our findings suggest that relatively healthy-to-slightly overweight dogs, distribute fat similarly between both their abdominal subcutaneous and peritoneal compartments. This finding may explain the confusion in the literature as to the specific importance of regional fat distribution relative to the overall fat percentage, in the pathogenesis of obesity-related diseases. Both overall obesity and intra-abdominal fat volume have been associated with similar findings of elevated serum leptin, insulin and inflammatory cytokine concentration, insulin resistance and cardiovascular dysfunction, which may be ascribed to the fact that both total body fat percentage and visceral fat volume appear directly related [2, 6, 7, 24, 25].

The results of this study are supported by other studies that have found a similar lack of relationship between overall fat percentage and V/SQ ratio [2, 19, 26]. A weakness in this study is the constrained range of body condition scores in our sample group, and our results should be cautiously extended to thin or obese dogs. Variation in the compartmental fat distribution has been documented in longitudinal obesity studies using Beagles, where there was no consistent change in the V/SQ ratio with changes in adiposity [2, 19]. The importance of V/SQ ratio in dogs is yet to be fully elucidated in metabolic diseases. Some studies found leptin secretion was positively correlated with overall body fat percentage and visceral fat, but not with V/SQ fat ratio, and proposed that leptin is predominantly secreted from visceral fat in dogs [7, 24, 25].

The primary determinant of V/SQ ratio in our study was age. To the authors’ knowledge, this is the first time this relationship has been documented. However, the relationship is not surprising given the findings in other studies that show total body adiposity correlates with increasing age [27].

Sex and neuter status may be other factors that influence the regional distribution of fat around the abdomen. In our small sample group, there was no difference in fat compartmentalisation (V/SQ) between male and female dogs; and the number of entire dogs in the sample were too small to make any inference. However, other studies have found a relationship, and the influence of sex and neuter status on body fat compartmentalisation is still plausible, but likely requires a larger sample size to discern it [28].

The impact of body conformation and breed disposition on fat distribution were not assessed in our study, but further investigation into this area may prove an interesting research opportunity. This suggestion stems from human research that has found that body types of regional fat distribution (android-gynoid fat ratio), combined with lean tissue mass, being contributory to metabolic health rather than just total body fat or intra-abdominal compartmentalisation of fat [29].

Other factors such as the effects of diet were not evaluated, however, either a greater study population or specific case-control study would be required, and further research into these relationships is encouraged. A case-control study looking at 48 Labrador retrievers fed either a controlled diet or a 25% restricted diet over the course of their life span found that the controlled diet consistently had greater body fat than the restricted diet, but both groups increased in body fat percentage with age [27]. Another study in beagles assessed the effects of glycaemic index on metabolic parameters, adiposity and fat distribution, found that neither a low or high glycaemic index food altered the distribution of V/SQ fat ratio over the course of the study [26]. Finally, higher protein diets fed after neutering may negate increasing body fat mass in dogs [28].

For our population of normal to slightly overweight dogs, the average V/SQ ratio was 0.6 (range: 0.36 to 0.86). A “normal” ratio has not been established; however studies assessing beagles, reported an average V/SQ ranging from 0.33–0.43 for 7–8 normal and obese dogs, but in one study it ranged from 0.29–2.64 [2, 19, 26]. The relatively wide range in some of these values may reflect methodological differences such as using segmental area or segmental volume versus en bloc volume used in our study, but also demonstrates other variations in V/SQ that may be useful to explore. Further, this variation could result in both veterinarians and researchers under- and over-estimating the potential for obesity-related diseases, when solely using total and superficial fat percentage estimators such as body condition scoring.

Computed tomography overcomes this subjectivity and is an excellent research tool to determine body composition, and regional and compartmental distribution of fat, however access to this modality is limited [2, 6, 7]. To overcome this, we have shown that the ultrasound measured visceral adipose thickness to subcutaneous adipose thickness (VAT/SAT) can provide a simple, more accessible means to estimate abdominal fat distribution in dogs. Further, that visceral adipose thickness (falciform fat) may be a useful means of evaluating intra-abdominal fat mass (visceral fat) in dogs (r = 0.832). This measurement could also be helpful in assessing falciform fat thickness if the premise is that this region is a preferential leptin secretor [24]. However, we did find the technique more challenging than we expected, and the method requires further optimisation to improve its reproducibility. A further consideration is that females had reduced VAT/SAT compared to male dogs, and likely reflects the removal of falciform fat during the ovariohysterectomy. Thus, further research using this method should take this into consideration.

As mentioned, a limitation to our study is the small sample group, which limits the power in determining the effects of the complex number of variables and confounders that may influence body composition and regional fat distribution. Related to this is the small range of body conditions scores, preventing inference of distribution of fat in dogs that are emaciated or obese. Another limitation we did not address is that complete body CT was not performed, which would have allowed regional fat distribution to be fully assessed such as appendicular and thoracic distribution.

## Conclusions

Our findings suggest that dogs with a relatively healthy to slightly overweight body condition score, distribute fat relatively similarly between their intra-abdominal and subcutaneous compartments with increasing total body fat percentage. However, there was increased fat distribution to the visceral compartment relative to the subcutaneous compartments with increasing age. Further, abdominal ultrasound may be useful in estimating the ratio of fat distribution to both the visceral and subcutaneous spaces.

## Methods

### Ethics

The University of Melbourne Faculty of Veterinary and Agricultural Sciences Animal Ethics Committee granted ethical approval (Ethics ID: 1613993).

### Animals

Twenty-two dogs of variable breeds were sourced from the staff and students of the U-Vet Werribee Animal Hospital, University of Melbourne. Dogs were excluded if systemically unwell. Dogs were fasted for 12 h, weighed and assigned a body condition score out of 9 as described previously [11] by one investigator (RT) on the morning of the imaging analysis. The same scales were used to weigh each dog and were tared daily.

### Experimental protocol

Dogs were sedated with intravenous medetomidine hydrochloride (10 μg/kg of body weight) and butorphanol (100 μg/kg of body weight), administered via the cephalic vein. Whole body DXA, abdominal CT acquisition and ultrasound fat measurements were performed sequentially and within 1 h of each other. The dogs were recovered and returned to their owners after the imaging studies. The study methodology was used in parallel research validating abdominal CT volume assessment to estimate body composition [17].

### Body composition estimation by dual-energy X-ray absorptiometry (DXA)

Body composition was estimated in a parallel study using a Hologic Discovery W dual-energy X-ray absorptiometer (Hologic, Waltham, MA, USA) [17]; DXA acquisition was performed in lateral recumbency and the total body fat (g) and total body fat percentage were estimated.

### Computed tomography (CT) and abdominal fat measurement

Volume acquisition of the abdomen was performed using a 16-slice CT scanner (Somatom Emotion 16, Siemens, Erlangen, Germany). CT data were stored in DICOM format and stored on the U-Vet Werribee Animal Hospital PACS. Proprietary software (Somaris/5 Syngo CT 2014A, Siemens AG, Muenchen, Germany) was used for semi-automated volume quantification of body composition and distribution.

As previously published, the abdominal volume of interest (VOI) was established between the cranial margin of the 10th thoracic vertebrae to the cranial margin of first sacral vertebrae [17]. Manual adjustments of the automated boundaries were performed to ensure the defined regions were maintained. The volume of tissue between these regions was automatically calculated by the software using Hounsfield threshold ranges of − 250/2000 HU for all tissues; and − 250/− 25 HU for fat, as validated in a prior study [17]. The fat mass was then calculated using an assumed fat density of 0.923 kg/L [30].

**Fig. 3:**
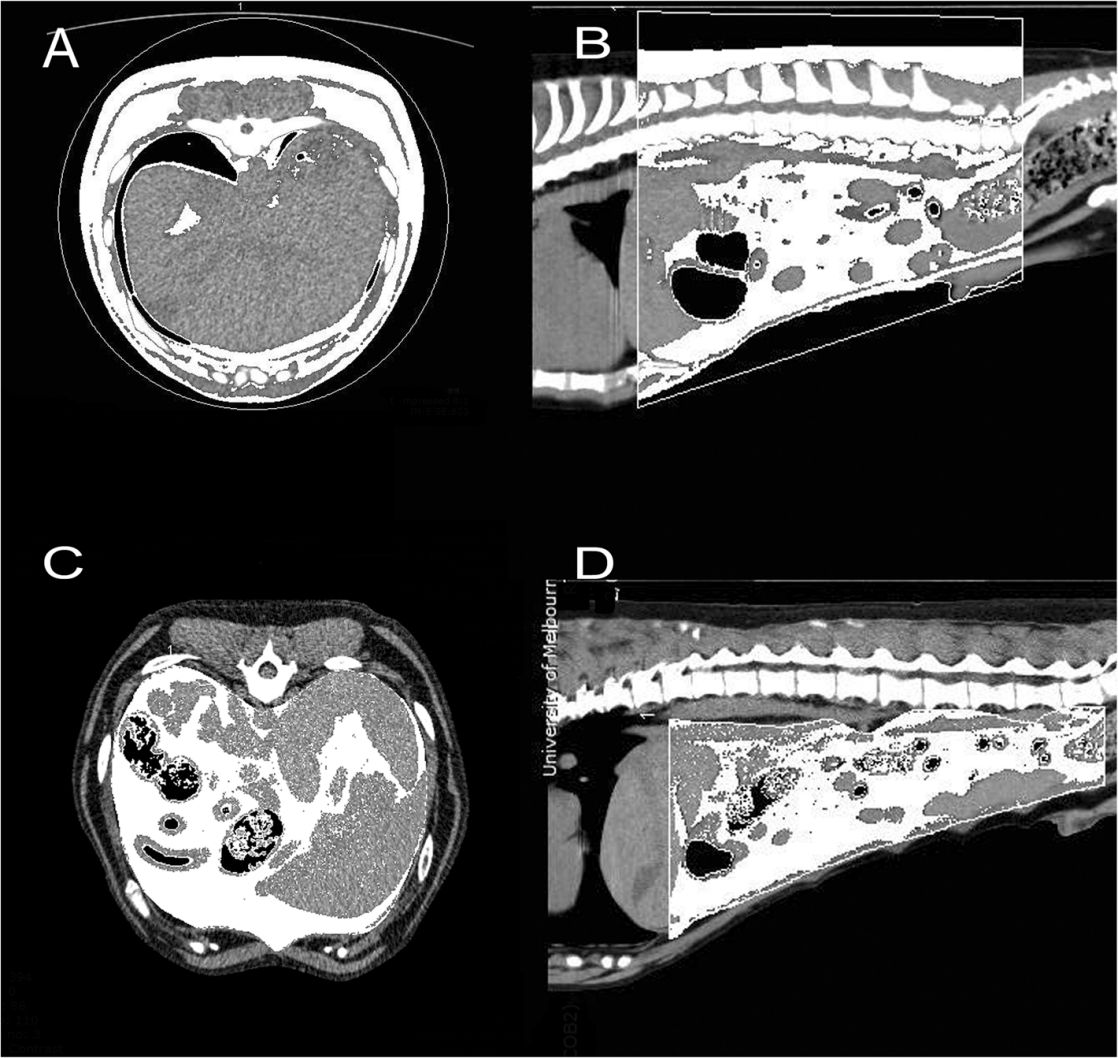
CT images of two dogs in transverse and sagittal planes showing volume segmentation of total abdominal fat (**a** and **b**) and the peritoneal fat (**c** and **d**) using thresholds of − 250 to -25HU

The total abdominal fat was defined as all fat within the fixed abdominal region. The visceral fat volume (V) was defined as fat within the intra-abdominal compartment of the peritoneal and retroperitoneal cavities. The circumferential boundaries of the intra-abdominal compartment were traced as free-hand ROIs along the ventral aspect of the included vertebrae, the inner surface of the included rib cage, the ventral margin of the lumbar hypaxial muscles, the visible peritoneum, and the inner boundaries of the rectus abdominis muscle and transversus abdominis muscle. This was modified from previous descriptions in dogs and humans, as the lumbar hypaxial musculature was excluded from the visceral compartment in this study [19, 26, 31]. The ROIs were drawn at the cranial and caudal margins of the total abdominal boundaries and repeated segmentally every one to two vertebral spaces to improve the VOI’s semi-automated boundaries. The semi-automated boundaries were manually inspected and adjusted if they deviated from the defined region (see Fig. 3).

The abdominal subcutaneous fat volume (SQ) was calculated by subtracting visceral fat volume from the total abdominal fat volume. Subcutaneous fat was inclusive of subcutaneous fat, inter-muscular and intramuscular fat, outside of the peritoneal cavity. The visceral and subcutaneous fat volumes were used to determine the volume visceral-to-subcutaneous fat ratio (V/SQ).

Further variables were calculated from the CT volumes including:

- Abdominal fat percentage (AB%) calculated as abdominal fat mass relative to total body mass.
- Relative abdominal fat percentage (rel AB%) calculated as abdominal fat mass relative to total body fat mass.
- Visceral fat percentage (V %) calculated as visceral fat mass in the fixed abdominal region relative to total body mass.
- Relative visceral fat percentage (rel V %) calculated as visceral fat mass in the fixed abdominal region relative to total body fat mass.
- Abdominal subcutaneous fat percentage (SQ %) calculated as subcutaneous fat mass around abdomen, relative to total body mass.
- Relative abdominal subcutaneous fat percentage (rel SQ %) calculated as subcutaneous fat mass around abdomen, relative to total body fat mass.

### Ultrasound measurements of subcutaneous and visceral fat thickness

**Fig. 4:**
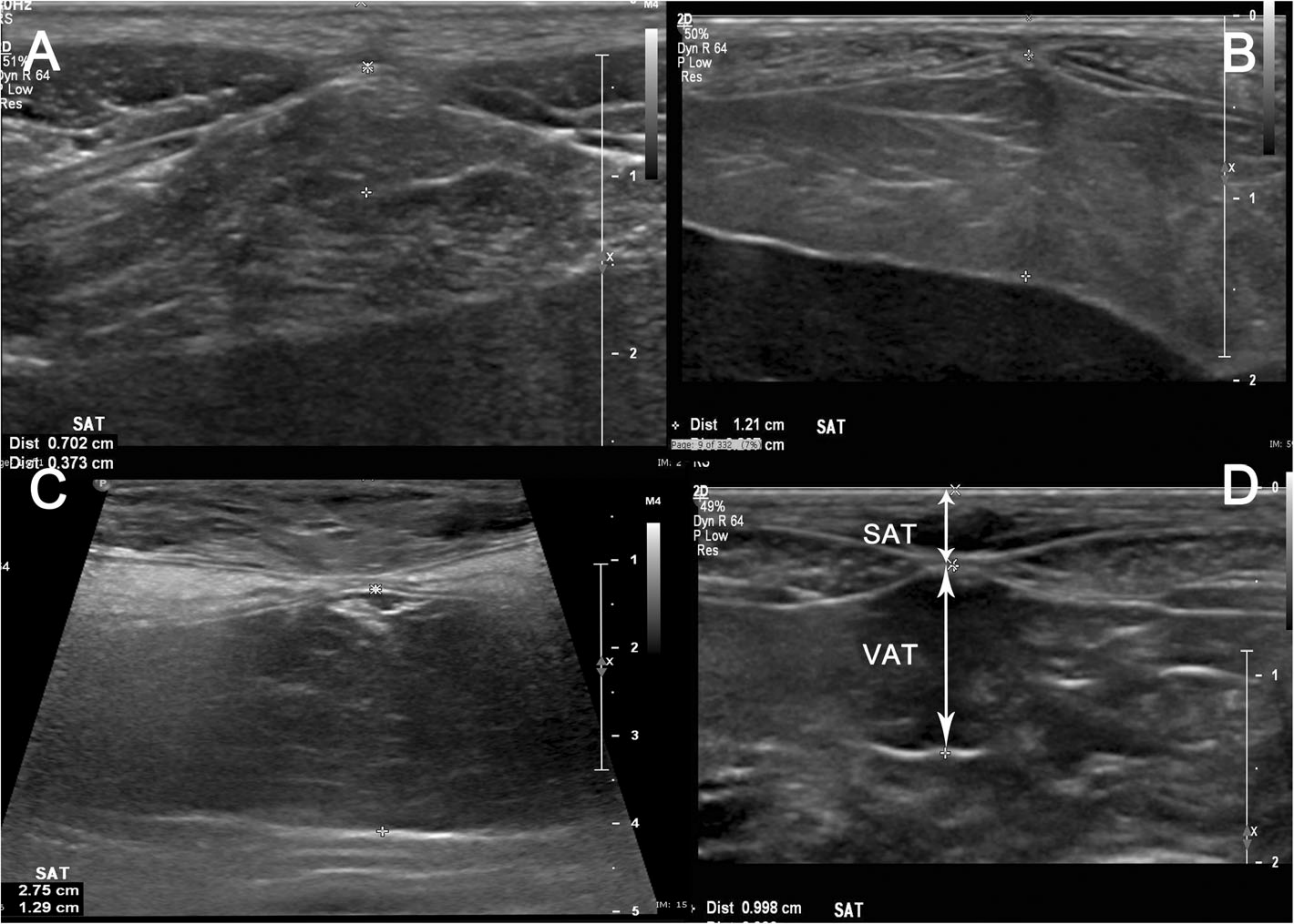
Ultrasound images of 4 dogs in dorsal recumbency showing the location of subcutaneous adipose thickness (VAT) and visceral adipose thickness (VAT) measurements. The transducer is located approximately 2-5 cm cranial to umbilicus in transverse plane to the linear alba

Ultrasound measurements were performed using a 15 MHz linear transducer (Philips EPIQ 5). The dogs were placed in dorsal recumbency, and all measurement performed three times by a single investigator (RT) and stored in DICOM format and stored on the U-Vet Werribee Animal Hospital PACS. The subcutaneous and visceral fat measurements were acquired on the ventral midline, 2–5 cm cranial to the umbilicus. Hair clipping was not required at this location, as there was adequate alcohol and ultrasound gel coupling. The ultrasound probe was repositioned and measurements performed a minimum of 3 times with minimal probe compression and the mean value used for calculations. The subcutaneous adipose thickness (SAT) was measured from the skin surface to the transversalis fascia/inner raphe of the rectus abdominis muscles. The visceral adipose thickness (VAT) was measured perpendicular to and from the transversalis fascia to the first major horizontal fascial believed to be the dorsal margin of the falciform fat. If this was not identifiable, the ventral surface of the adjacent organ was used. All measurements were performed in full expiration and repeated at peak inspiration (VAT_insp_) in a subset of dogs (*n* = 11). The total adipose thickness (TAT) was defined as the addition of VAT and SAT at the same site (see Fig. 4).

### Reliability of ultrasound measurements

Inter- and intra-observer variability of determining CT abdominal fat volumes was established in a previous study [17]. Intra-class correlation (ICC) and 95% confidence intervals were used to assess both intra- and inter-observer agreement. Due to scheduling conflicts between the two observers, a limited sample of 5 dogs was used. For intra-observer variability, each observer performed 3 separate measurements on each dog. The transducer was removed and repositioned after each measurement and the observers were blinded to each other’s measurements. For inter-observer variability, the average of the observers’ three measurements for each animal were compared. The ICC was calculated for VAT, SAT, TAT and VAT/SAT measurements.

### Statistical methods

The sample size was determined by the inclusion of the dogs in another validation study [17]. As experimental method validation was being assessed, a minimum of 20 samples, with a rolling sample size to 40 was sought [32].

Relationships between variables were visualised on scatter plots and assessed using linear correlation or independent samples t-tests. The assumption of normality was evaluated and found to be adequately satisfied. Two-tailed *p*-values were used, and p-values less than 0.05 were considered statistically significant. For statistically significant results, the sample means (M), and sample standard deviations (SD) were reported, with the 95% confidence interval of the difference of the means (95CI) and the Cohen’s effect size value (d) [33]. A standard interpretation of Cohen’s effect size of small effect (d = 0.2), medium effect (0.5) and large effect (0.8) was used. The correlation coefficient for continuous variables was described as perfect (r = 1.00) very strong (> 0.90), strong (0.70–0.90), moderate (0.50–0.70), poor (0.30–0.50) and weak (0.00–0.30) correlation [34]. Multiple linear regression was performed to model the influence of predictor variables on AB% and the visceral/subcutaneous fat ratio (V/SQ), using predictor variables with a reasonably linear relationship (r ≥ 0.3). Additionally, as the scales of the independent variables differed, the standardised coefficients (β) were reported. Linear models were fitted to determine the effect of sex (male and female) on either total abdominal fat percentage and V/SQ, whilst controlling for DXA total body fat percentage and age. Levene’s test and normality checks were carried out and the assumptions adequately met. A Bonferroni adjustment was used and the adjusted R square value (R^2^_Adjusted_) reported for the models. Desexing status (neutered and entire dogs) was excluded from the analysis due to the small sample number of entire dogs (*n* = 4). Statistical analysis was performed using GraphPad Prism (GraphPad Prism for Mac OS X, version 7.0c, GraphPad Software, La Jolla, CA, USA, www.graphpad.com), and SPSS (IBM SPSS Statistics for Mac, version 25, IBM Corporation, Armonk, NY, USA).

## Data Availability

The datasets used and/or analysed during the current study are available from the corresponding author on reasonable request.

## Abbreviations

AB: abdominal fat volume (as measured by CT)
BCS: body condition score
CL: confidence limit
CT: computed tomography
CV: coefficient of variation
DXA: dual energy x-ray absorptiometry
HU: Hounsfield units
ROI: region of interest
SAT: subcutaneous adipose tissue thickness (as measured by ultrasound)
SQ: abdominal subcutaneous fat volume (as measured by CT)
TAT: total adipose tissue thickness (as measured by ultrasound = VAT+SAT)
V: visceral fat volume (as measured by CT)
VAT: visceral adipose tissue thickness (as measured by ultrasound)
VOI: volume of interest
V/SQ: visceral-to-subcutaneous fat ratio as measured by CT
